# Cobicistat-boosted darunavir in HIV-1-infected adults: week 48 results of a Phase IIIb, open-label single-arm trial

**DOI:** 10.1186/1742-6405-11-39

**Published:** 2014-12-01

**Authors:** Karen Tashima, Gordon Crofoot, Frank L Tomaka, Thomas N Kakuda, Anne Brochot, Tom Van de Casteele, Magda Opsomer, William Garner, Nicolas Margot, Joseph M Custodio, Marshall W Fordyce, Javier Szwarcberg

**Affiliations:** The Miriam Hospital, Alpert Medical School of Brown University, 164 Summit Avenue, Providence, RI 02906 USA; Gordon Crofoot Research, Houston, TX USA; Janssen Research & Development LLC, Titusville, NJ USA; Janssen Research & Development, Beerse, Belgium; Janssen Infectious Diseases BVBA, Beerse, Belgium; Gilead Sciences, Foster City, CA USA

**Keywords:** Cobicistat, Darunavir, Safety, Efficacy, Virology, Pharmacokinetics

## Abstract

**Background:**

Cobicistat is an alternative pharmacoenhancer to ritonavir. In healthy volunteers, darunavir exposure was comparable when darunavir 800 mg once daily was co-administered with cobicistat 150 mg once daily (as single agents or a fixed-dose combination) vs. with ritonavir 100 mg once daily.

**Methods:**

This 48-week, Phase IIIb, single-arm, US multicenter study (NCT01440569) evaluated safety, efficacy and pharmacokinetics of darunavir/cobicistat 800/150 mg once daily (as single agents) plus two investigator-selected nucleoside/tide reverse transcriptase inhibitors (N[t]RTIs) in HIV-1-infected adults. Patients had no darunavir resistance-associated mutations (RAMs), plasma viral load (VL) ≥1000 HIV-1 RNA copies/ml, eGFR ≥80 ml/min and genotypic sensitivity to the two N[t]RTIs. The primary endpoint was any treatment-emergent grade 3 or 4 adverse events (AEs) through Week 24.

**Results:**

The majority of the 313 intent-to-treat patients were treatment-naïve (295/313; 94%), male (89%), White (60%) and received a tenofovir-based regimen (99%). Median baseline VL and CD4^+^ count overall were 4.8 log_10_ HIV-1 RNA copies/ml and 361 cells/mm^3^, respectively. Overall, 86% of patients (268/313) completed the study. The majority of discontinuations were for AEs (15/313; 5%). The incidence of treatment-emergent grade 3 or 4 AEs regardless of causality was 6% through Week 24 and 8% through Week 48. Most common AEs through Week 48 were diarrhea (27%) and nausea (23%), which were grade 1 or 2 in severity. Week 48 virologic response rates (% with VL <50 HIV-1 RNA copies/ml; Snapshot analysis) were 81% overall and 83% in treatment-naïve patients; median increases in CD4^+^ count at 48 weeks were 167 and 169 cells/mm^3^, respectively. Of 15/313 patients who met the criteria for resistance analysis, one developed a darunavir RAM as a mixture with wild-type (I84I/V), without phenotypic resistance to darunavir. The mean population pharmacokinetic-derived darunavir areas under the plasma concentration–time curve were 102,000 overall and 100,620 ng•h/ml in treatment-naïve patients. No clinically relevant relationships were seen between darunavir exposure and virologic response, AEs or laboratory parameters.

**Conclusion:**

Darunavir/cobicistat 800/150 mg once daily was generally well tolerated through Week 48, with no new safety concerns. Pharmacokinetics, virologic and immunologic responses for darunavir/cobicistat were similar to previous data for darunavir/ritonavir 800/100 mg once daily.

## Introduction

Treatment guidelines for HIV-1 infection [1–4] include the recommendation of a ritonavir-boosted protease inhibitor (PI), such as darunavir/ritonavir, in combination with other antiretrovirals. In two randomized, controlled Phase III trials, darunavir/ritonavir 800/100 mg once daily demonstrated antiviral efficacy with long-term tolerability in treatment-naïve (ARTEMIS; TMC114-C211) [5–7] and treatment-experienced patients with no darunavir resistance-associated mutations (RAMs) (ODIN; TMC114-C229) [8].

Low-dose ritonavir (100 mg once daily or twice daily) is used as a pharmacokinetic enhancer [9]. Ritonavir is a potent inhibitor of cytochrome P450 (CYP) 3A, and thereby increases the oral bioavailability of most HIV-1 PIs, including darunavir [9, 10]. Low-dose ritonavir is associated with gastrointestinal disorders, such as diarrhea and nausea [9], hyperlipidaemia [11], and also clinically significant drug–drug interactions [12].

Cobicistat (GS-9350) is also a potent inhibitor of CYP3A and a pharmacoenhancer [13–16]. Cobicistat has no antiviral activity, does not induce CYP isozymes, and is more selective than ritonavir in terms of CYP3A inhibition [13, 14]. Cobicistat can be coformulated into fixed-dose combinations [17], thereby reducing pill burden and medication errors [18–22]. Cobicistat has been evaluated as part of a single-tablet regimen with elvitegravir, emtricitabine and tenofovir in clinical studies of HIV-1-infected, antiretroviral treatment-naïve adults [15, 16, 23–28]. Cobicistat 150 mg once daily for 144 weeks was generally well tolerated. Small decreases in estimated glomerular filtration rate calculated using the Cockcroft-Gault method (eGFR_CG_) were observed with cobicistat in these studies, which are attributable to inhibition of the tubular secretion of creatinine leading to creatinine increases as early as Week 2 and stabilizing by Week 24 through Week 144, without affecting renal function (actual GFR, aGFR) as measured by iohexol renal clearance [29].

A fixed-dose formulation of darunavir/cobicistat has been developed. A Phase I study demonstrated comparable darunavir pharmacokinetic parameters following darunavir 800 mg once daily co-administered with cobicistat 150 mg once daily, either as single agents [30] or as two candidate fixed-dose combination formulations [31] to those of darunavir/ritonavir 800/100 mg once daily. Bioequivalence of darunavir administered as a fixed-dose combination with cobicistat vs. single agents, was established under fasted or fed conditions [32].

The aim of this Phase IIIb study was to evaluate the safety, tolerability, efficacy and pharmacokinetics of darunavir in combination with cobicistat (as single agents) with a background regimen of two, fully active, investigator-selected nucleoside/tide reverse transcriptase inhibitors (N[t]RTIs) in HIV-infected treatment-naïve and -experienced adults with no darunavir RAMs.

## Results

### Patient disposition and baseline characteristics

The first patient in the study began treatment on October 24 2011, and the last patient in the study had their Week 48 visit on January 31 2013.

**Figure 1:**
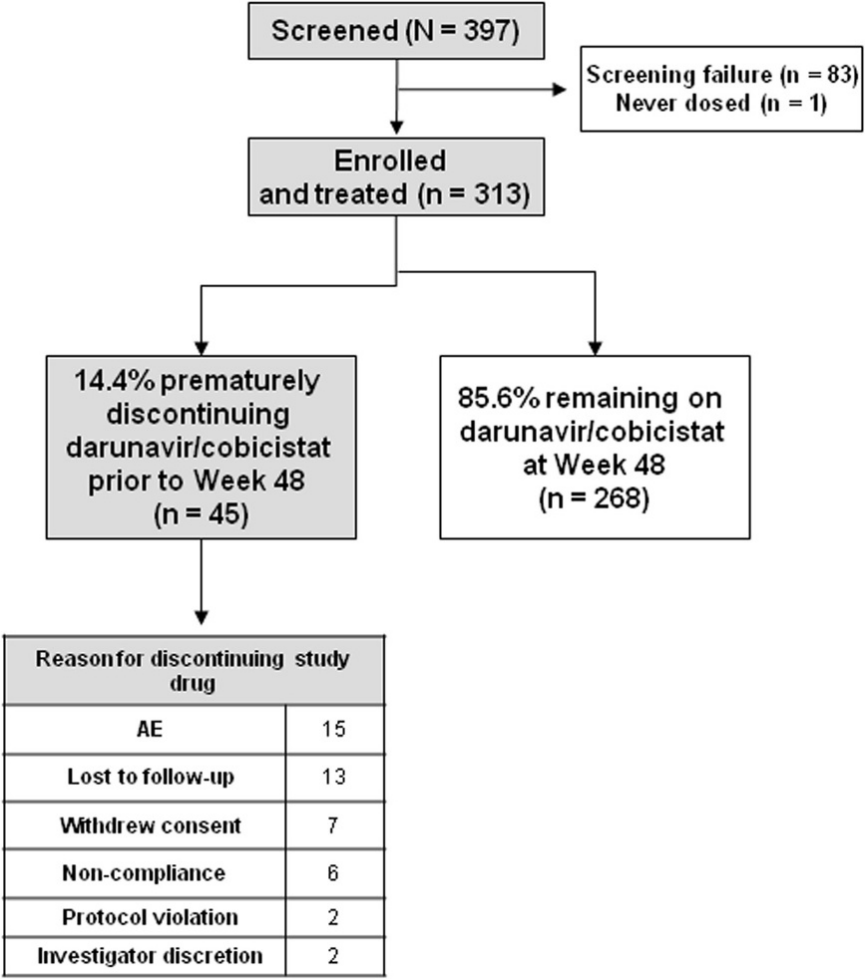
**Patient disposition through 48 weeks in the overall population.**

Of the 397 patients screened, 313 were enrolled and included in the intent-to-treat (ITT) population (Figure 1). One patient was not treated. Most of the 83 screening failures were due to screening eGFR_CG_ <80 ml/min or screening viral load (VL) <1000 HIV-1 RNA copies/ml. Of the 313 ITT patients, 295 were treatment-naïve and 18 were treatment-experienced with no darunavir RAMs. Overall, 86% of the ITT patients (268/313) completed the study and 14% (45/313) discontinued, mainly for AEs (n =15) and loss to follow-up (n =13) (Figure 1).

Overall, most patients were male (89%) and were White (60%) or Black or of African Heritage (35%). The median age was 35 years (range 18 to 72 years) (Table 1). Median (range) baseline VL and CD4^+^ cell count were 4.8 (2.6 to 7.0) log_10_ HIV-1 RNA copies/ml and 361 (5 to 1473) cells/mm^3^, respectively. Overall, 2% of patients were co-infected with hepatitis B and 3% with hepatitis C virus. Baseline characteristics of the treatment-naïve patient population are shown in Table 1.

**Table 1:** **Patient baseline demographics and disease characteristics and background N[t]RTIs**

	All patients N = 313	Treatment-naïve patients N = 295
Male [n (%)]	279 (89)	266 (90)
Age, years [median (range)]	35 (18–72)	34 (18–72)
Race [n (%)]		
White	187 (60)	176 (60)
Black or African Heritage	108 (35)	101 (34)
Asian	4 (1)	4 (1)
Other	14 (4)	14 (5)
Log_10_ VL (HIV-1 RNA copies/ml) [median (range)]	4.8 (2.6–7.0)	4.8 (2.6–7.0)
VL >100,000 HIV-1 RNA copies/ml [n (%)]	131 (42)	122 (41)
CD4^+^ cell count (cells/mm^3^) [median (range)]	361 (5–1473)	370 (6–1473)
≤200 cells/mm^3^ [n (%)]	59 (19)	47 (16)
Asymptomatic HIV infection [n (%)]	251 (80)	241 (82)
Co-infection with HBV [n (%)]	5 (2)	5 (2)
Co-infection with HCV [n (%)]	8 (3)	7 (2)
eGFR_CG_, ml/min [median (range)]	114 (67–321)	115 (67–321)
Background N[t]RTIs [n (%)]		
Emtricitabine/tenofovir	301 (96)	291 (99)
Emtricitabine/tenofovir plus zidovudine	5 (2)	0
Abacavir plus tenofovir	3 (1)	2 (0.7)
Emtricitabine/tenofovir plus abacavir	2 (0.6)	1 (0.3)
Abacavir/lamivudine	1 (0.3)	1 (0.3)
Didanosine plus emtricitabine	1 (0.3)	0

*N* number of patients; *n* number of patients with observations; *VL* viral load; *HBV* hepatitis B virus; *HCV* hepatitis C virus; *eGFR*_*CG*_ estimated glomerular filtration rate calculated using the Cockcroft-Gault method; N[t]RTIs nucleoside/tide reverse transcriptase inhibitors.

At baseline, one or more primary PI RAMs were found in 10 patients (3%; nine treatment-naïve, and one treatment-experienced), most commonly M46I/L (three treatment-naïve and one treatment-experienced) and Q58E (four treatment-naïve). No patients had darunavir RAMs. Secondary PI RAMs were found in 96% of patients (300/313; 283 and 17 patients), reflecting the strong polymorphic nature of these mutations. Non-nucleoside reverse transcriptase inhibitor (NNRTI) RAMs were found in 28% of patients (87/313; 74 and 13 patients), most commonly K103N/S (13%; 41/313; 33 and eight patients). N[t]RTI RAMs were found in 14% of patients (43/313; 36 and seven patients), most commonly V118I (6%; 18/313; 18 and zero patients), T69D/N (3%; 8/313; eight and zero patients) and M184V/I (3%; 8/313; two and six patients).

### Concomitant N[t]RTI use

The majority of patients (99%; 311/313 overall and 294/295 treatment-naïve patients) received tenofovir-based antiretroviral therapy during the study. The most frequently used N[t]RTI combination was emtricitabine/tenofovir (Table 1).

### Adherence

During the course of the study, median adherence to darunavir and cobicistat in the overall population through Week 48 as measured by pill count was 100%, and 299/313 patients (96%) had a ≥90% adherence rate. Median adherence to darunavir and cobicistat in the treatment-naïve population through Week 48 as measured by pill count was also 100%, and 283/295 patients (96%) had a ≥90% adherence rate.

### Safety and tolerability

The overall median duration of exposure to study drugs was 64.3 weeks (58.3 to 69.3 weeks); 268/313 (86%) patients received the study drugs for ≥48 weeks.

The most commonly reported treatment-emergent AEs regardless of causality during the study were diarrhea, nausea, upper respiratory tract infection and headache (Table 2). The most common study drug-related AEs through Week 48 were diarrhea (15%; 47/313 overall and 15%; 43/295 treatment-naïve patients), nausea (14%; 45/313 and 15%; 44/295, respectively), headache (4%; 13/313 and 4%; 12/295) and flatulence (4%; 13/313 and 4%; 13/295).

**Table 2:** **Treatment-emergent AEs and grade 3–4 laboratory abnormalities**

	All patients		Treatment-naïve patients
Incidence, [n (%)]	Week 24 analysis	Week 48 analysis	Week 48 analysis
	N = 313	N = 313	N = 295
Grade 3–4 AEs	18 (6)	24 (8)	21 (7)
Any AE	275 (88)	286 (91)	270 (92)
Any drug-related AE	123 (39)	128 (41)	122 (41)
Serious AEs	15 (5)	26 (8)	21 (7)
Deaths	0	0	0
**AEs any grade, regardless of relationship to study treatment and occurring in ≥10% of patients**			
Diarrhea	78 (25)	86 (27)	80 (27)
Nausea	67 (21)	72 (23)	69 (23)
Upper respiratory tract infection	31 (10)	44 (14)	43 (15)
Headache	29 (9)	38 (12)	35 (12)
AEs leading to discontinuation*			
Any, n	15 (5)	16 (5)	16 (5)
Maculo-papular rash	3 (1)	3 (1)	3 (1)
Rash	3 (1)	3 (1)	3 (1)
Nausea	2 (0.6)	2 (0.6)	2 (0.7)
Hypersensitivity	2 (0.6)	2 (0.6)	2 (0.7)
Idiopathic thrombocytopenic purpura	1 (0.3)	1 (0.3)	1 (0.3)
Dyspepsia	1 (0.3)	1 (0.3)	1 (0.3)
Vomiting	1 (0.3)	1 (0.3)	1 (0.3)
Mycobacterium avium complex infection	1 (0.3)	1 (0.3)	1 (0.3)
Headache	1 (0.3)	1 (0.3)	1 (0.3)
Dysgeusia	1 (0.3)	1 (0.3)	1 (0.3)
Renal tubular disorder	–	1 (0.3)	1 (0.3)
Allergic dermatitis	1 (0.3)	1 (0.3)	1 (0.3)
Macular rash	1 (0.3)	1 (0.3)	1 (0.3)
Vesicular rash	1 (0.3)	1 (0.3)	1 (0.3)
**Treatment-emergent grade 3–4 laboratory abnormalities occurring in ≥2% patients**			
	**n = 310**	**n = 310**	**n = 292**
Increased creatine kinase	18 (6)	22 (7)	22 (8)
Increased alanine amino transferase	7 (2)	9 (3)	9 (3)
Increased aspartate amino transferase	6 (2)	8 (3)	7 (2)
Increased amylase	6 (2)	8 (3)	7 (2)
Increased lipase	5 (2)	7 (2)	7 (2)

*N* number of patients; *n* number of patients with observations; *AE* adverse event. Serious AEs included any AE that occurred at any dose that resulted in death, a life-threatening situation, inpatient hospitalization, persistent or significant disability/incapacity, congenital anomaly/birth defect in the offspring of a patient who received investigational medicinal product; *Patients may have discontinued due to more than 1 AE.

Most adverse events (AEs) were grade 1 or 2 in severity. The incidence of any grade 3 or 4 treatment-emergent AEs regardless of causality through Week 24 (primary endpoint) was low (Table 2). Overall, 16 patients experienced grade 3 (5%) and two patients experienced grade 4 (0.6%) AEs. Among the treatment-naïve patients, through Week 48, the most common grade 3 or 4 AEs regardless of causality (occurring in ≥2 patients) were hypersensitivity (grade 3: two patients; grade 4: one patient), maculo-papular rash (grade 3: two patients) and peripheral neuropathy (grade 3: two patients). Any study drug-related grade 3 AE occurred in five patients (2%). These grade 3 AEs were immune reconstitution syndrome (one patient), hypersensitivity (two patients), maculo-papular rash (one patient), and allergic dermatitis, maculo-papular rash and vesicular rash (all occurring in the same patient), and all led to study drug discontinuation. One patient had grade 4 thrombocytopenic purpura, which was considered serious and led to study drug discontinuation, but was not considered related to study drug. Another patient had grade 4 hypersensitivity, which was considered serious and led to study drug interruption, but was not considered related to study drug, but related to concomitant lisinopril.

Three serious AEs, all occurring in treatment-naïve patients, were felt to be related to study drug. One patient each reported immune reconstitution syndrome, rash and maculo-papular rash. There were no deaths. The most commonly reported AEs that led to discontinuation through Week 48 were rash and maculo-papular rash, nausea and hypersensitivity (Table 2). All these AEs were felt to be study drug-related and resolved upon discontinuation. One treatment-naïve patient discontinued because of renal tubular disorder, which was mild in severity, not serious and resolved following change in therapy to darunavir/ritonavir plus lamivudine and abacavir. Eight bone fractures were reported through Week 48, each with traumatic mechanisms and without features concerning for fragility fracture.

There was an increase in serum creatinine level from baseline occurring as early as Week 2 (median change = 0.10 mg/dl overall and in treatment-naïve patients), which remained stable throughout the Week 48 treatment period (median change =0.09 mg/dl and 0.08 mg/dl, respectively) and is consistent with cobicistat inhibition of creatinine secretion. There were no other clinically relevant changes from baseline in median values for other clinical laboratory parameters.

### Virologic response

**Figure 2:**
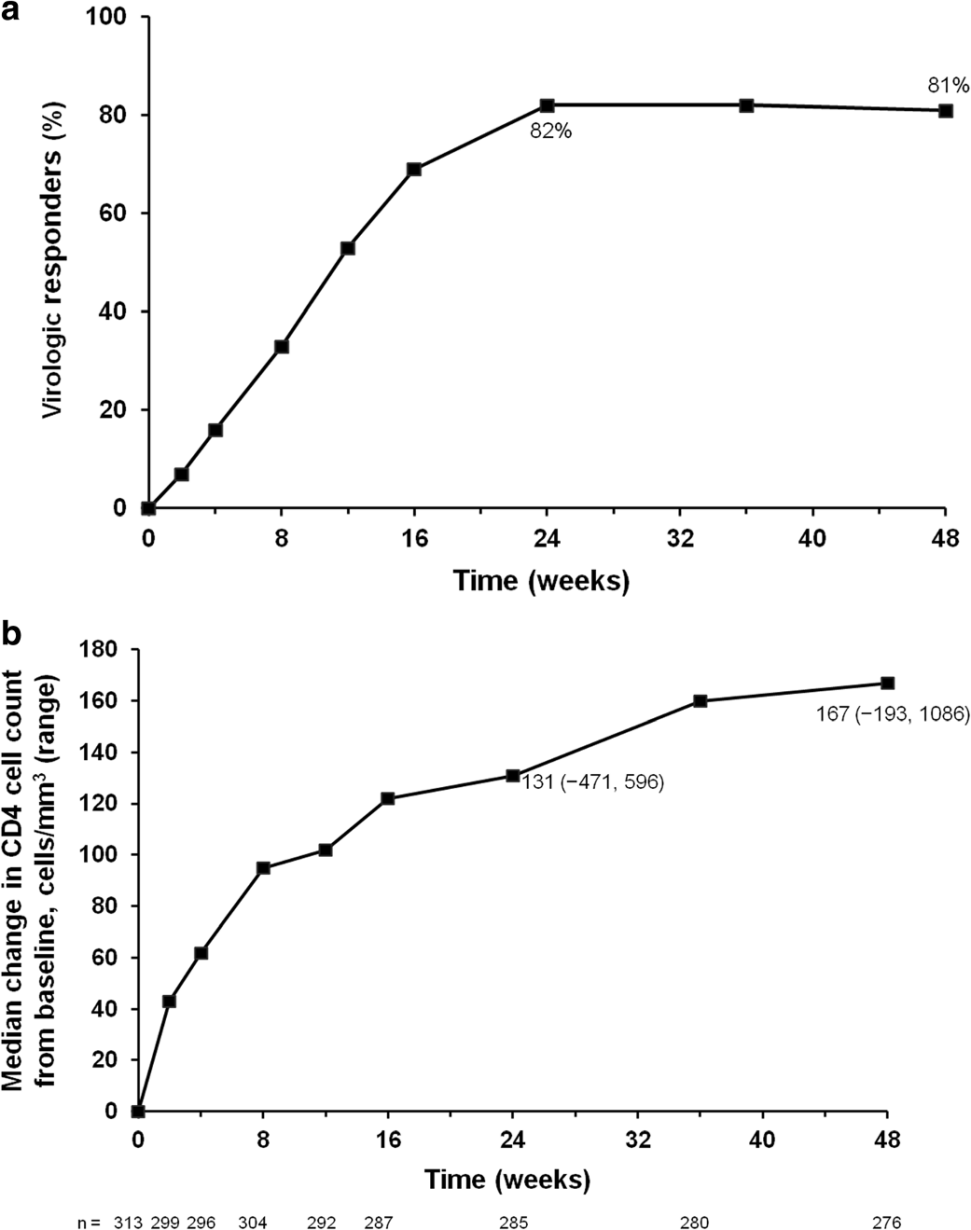
**Efficacy through 48 weeks in the overall population. (a)** Proportions of viral load responders (<50 copies/ml: FDA snapshot analysis) through 48 weeks (N =313); **(b)** Median (range) change in CD4^+^ cell count (cells/mm^3^) from baseline through 48 weeks (missing = excluded).

Overall, the virologic response rate (Food and Drug Administration [FDA] snapshot analysis) was 82% (258/313) (95% confidence interval [CI] 78%, 87%) at Week 24 and was sustained and durable through Week 48 (81%; 95% CI 76%, 85%; Table 3 and Figure 2). Virologic failure occurred in 11% of patients at Week 48, and 9% had no virologic data in the Week 48 window.

**Table 3:** Virologic outcome at Week 24 and 48 according to the FDA Snapshot* analysis in the overall population

	Week 24			Week 48		
Outcomes, [n (%)]		VL ≤100 K HIV-1	VL >100 K HIV-1		VL ≤100 K HIV-1	VL >100 K HIV-1
	Total	RNA copies/ml	RNA copies/ml	Total	RNA copies/ml	RNA copies/ml
	n = 313	n = 182	n = 131	n = 313	n = 182	n = 131
**Virologic response**	**258 (82)**	**157 (86)**	**101 (77)**	**253 (81)**	**148 (81)**	**105 (80)**
**Virologic failure**	**36 (12)**	**10 (5)**	**26 (20)**	**33 (11)**	**14 (8)**	**19 (15)**
VL ≥50 HIV-1 RNA copies/ml	22 (7)	4 (2)	18 (14)	14 (4)	5 (3)	9 (7)
Discontinued drug due to lack of efficacy	0	0	0	0	0	0
Discontinued drug due to other reasons and last available VL ≥50 HIV-1 RNA copies/ml	14 (4)	6 (3)	8 (6)	19 (6)	9 (5)	10 (8)
**No virologic data in the analysis window**	**19 (6)**	**15 (8)**	**4 (3)**	**27 (9)**	**20 (11)**	**7 (5)**
Discontinued drug to AE	14 (4)	12 (7)	2 (2)	15 (5)	13 (7)	2 (2)
Discontinued drug for other reason and last VL <50 HIV-1 RNA copies/ml	3 (1)	2 (1)	1 (1)	10 (3)	5 (3)	5 (4)
Missing data during window but on drug	2 (1)	1 (1)	1 (1)	2 (1)	2 (1)	0

*Proportion of patients with VL <50 HIV-1 RNA copies/ml during Week 24 or Week 48 window; *N* number of patients; *n* number of patients with observations; *AE* adverse event, *VL* viral load.

In treatment-naïve patients, the Week 48 virologic response rate was 83% (244/295; 95% CI 78%, 87%). Twenty four treatment-naïve patients (8%) were classed as virologic failures, and 27 treatment-naïve patients (9%) had no virologic data in the Week-48 window.

The Week 48 rates of virologic response were comparable in patients who had a baseline VL ≤100,000 HIV-1 RNA copies/ml or >100,000 HIV-1 RNA copies/ml, both overall (81% vs. 80%, respectively) (Table 3) and in treatment-naïve patients (84% vs. 81%).

Sensitivity analyses showed the Week 48 virologic response rate was 81% (253/313) overall and 83% (245/295) in treatment-naïve patients using the time-to-loss of virologic response (TLOVR) analysis, and it was 83% (260/313) and 85% (250/295), respectively, using the missing = failure (M = F) method.

### Immunologic response

There was an increase in CD4^+^ cell count from baseline at all timepoints following initiation of study drugs. At Weeks 24 and 48 in the overall population, the median (range) increases from baseline in CD4^+^ cell count (missing = excluded) were 131 (-471 to 596) cells/mm^3^ and 167 (-193 to 1086) cells/mm^3^, respectively (Figure 2). In treatment-naïve patients, the median (range) CD4^+^ cell count increased by 169 (-193 to 1086) cells/mm^3^ from baseline to Week 48.

### Development of resistance

Of the 15 patients with samples eligible for resistance analysis (Table 4), three had suboptimal virologic response, eight had virologic rebound, and four who discontinued with VL ≥400 HIV-1 RNA copies/ml were analyzed at their last visit. Only one of these 15 patients who was treatment experienced (prior antiretrovirals included efavirenz, emtricitabine, tenofovir disoproxil fumarate, zidovudine, lamivudine, stavudine) developed a resistance mutation to darunavir (at position I84 as a mixture with wild-type, I84I/V) (Table 4); this was not associated with phenotypic resistance to darunavir or other PIs. Two patients (one treatment-experienced and one treatment-naïve) developed the M184V N[t]RTI RAM in reverse transcriptase while receiving emtricitabine (Table 4) that was associated with phenotypic resistance to emtricitabine and lamivudine. One patient (patient 14 in Table 4) showed the transient development of the N[t]RTI RAM L74I/L and the NNRTI RAM P225H/P at the Week 16 visit, which were not detected at the subsequent Week 48 visit analysis as shown in Table 4. These mutations were not associated with resistance to the agents in the patient’s regimen (emtricitabine/tenofovir/zidovudine), and may reflect previous drug history. New primary RAMs were not detected in the 11 remaining patients.

**Table 4:** **Post-baseline genotypic analysis through Week 48 in the 15 patients in the resistance analysis population**

Patient number	N[t]RTI backbone	Treatment cohort	Visit (week)	RAMs emerging*	
				Protease	Reverse transcriptase
1	Abacavir plus tenofovir	Naïve	24	None	None
2	Emtricitabine/tenofovir	Naïve	12	None	None
3	Emtricitabine/tenofovir	Naïve	48	I93I/L	I142L/P
4	Emtricitabine/tenofovir	Experienced	24	None	K22K/R
5	Emtricitabine/tenofovir	Naïve	36	R57R/K	**M184V**
6	Emtricitabine/tenofovir	Naïve	16	V32V/A, P39P/S	K122K/E, D123D/G/N/S, D177D/G, I178M
7	Emtricitabine/tenofovir	Naïve	48	None	D123D/E
8	Emtricitabine/tenofovir	Experienced	48	None	K122K/E, A272A/P
9	Emtricitabine/tenofovir	Experienced	12	**I84I/V**	E138T, T165A/E, R211R/K
10	Emtricitabine/tenofovir	Naïve	8	G86G/E	D123N
11	Emtricitabine/tenofovir plus zidovudine	Experienced	48	None	I195I/L
12	Abacavir plus tenofovir	Experienced	36	R41R/K	None
13	Emtricitabine/tenofovir	Naïve	48	V77V/I	E6E/K, E224E/Q, V245Q, T286T/A, V292V/I
14	Emtricitabine/tenofovir plus zidovudine	Experienced	48	None	E6E/D, V60I
15	Emtricitabine/tenofovir	Experienced	36	None	**M184V**

*Compared to screening genotype – emerging primary resistance-associated mutations (RAMs) are shown in bold; *N[t]RTI* nucleoside/tide reverse transcriptase inhibitor.

### Pharmacokinetics

Sixty patients were enrolled in the pharmacokinetic substudy. Pharmacokinetic parameters were comparable to historic data. For darunavir, mean (standard deviation [SD]) maximum plasma concentration (C_max_) was 7663 (1920) ng/ml, minimum plasma concentration (C_min_) was 1310 (969) ng/ml and area under the concentration–time curve during a 24-hour interval (AUC_24h_) was 81,646 (26,322) ng•h/ml (Table 5). Pharmacokinetics of cobicistat, tenofovir and emtricitabine are shown in Table 5.

**Table 5:** **Summary of pharmacokinetic parameters in the substudy**

Pharmacokinetic parameters	Darunavir	Cobicistat	Emtricitabine	Tenofovir
n = 60				
AUC_tau_, ng•h/ml [mean (SD)]	81,646 (26,322)*	7596 (3657)*	11,793 (3490)^†^	3613 (1203)^†^
C_max_, ng/ml [mean (SD)]	7663 (1920)	991 (331)	1862 (491)*	382 (118)*
C_tau_, ng/ml [mean (SD)]	1311 (969)*	33 (95)*	113 (101)^†^	78 (33)^†^
C_0h_, ng/ml [mean (SD)]	1560 (1328)	76 (186)	147 (171)*	84 (45)*
T_max_, h [median (Q1, Q3)]	3.50 (2.49, 4.29)	3.50 (2.01, 4.50)	2.02 (2.00, 3.50)*	2.00 (1.00, 3.05)*
t_1/2_, h [median (Q1, Q3)]	7.24 (5.35, 11.54)^‡^	3.25 (2.91, 3.81)*	7.14 (6.44, 7.86)^§^	13.34 (11.68, 15.35)^§^

*n = 59; ^†^n = 58; ^‡^n = 55; ^§^n = 56.

In the Bayesian feedback analysis for darunavir population pharmacokinetic parameters through Week 48 (n = 298), the overall mean (SD) population pharmacokinetic-derived darunavir AUC_24h_ and trough plasma concentration (C_0h_; n = 298) at Week 48 were 102,000 (33,100) ng•h/ml and 2150 (1320) ng/ml, respectively. Data were available for 281 treatment-naïve patients. The mean (SD) darunavir AUC_24h_ was 100,620 (32,366) ng•h/ml and C_0h_ was 2105 (1289) ng/ml. In 17 treatment-experienced patients, the mean (SD) darunavir AUC_24h_ was 119,747 (39,961) ng•h/ml and C_0h_ was 2917 (1658) ng/ml. These values are comparable to those previously reported for darunavir/ritonavir 800/100 mg once daily (mean [SD] AUC_24h_ and C_0h_ were 93,026 [27,050] ng•h/ml and 2282 [1168] ng/ml, respectively, in antiretroviral-naïve patients [33] and 93,334 [28,626] ng•h/ml and 2160 [1201] ng/ml, respectively, in treatment-experienced patients with no darunavir RAMs [34]). There were no clinically relevant relationships between darunavir exposure and virologic response, AEs or laboratory parameters.

## Discussion

This Phase IIIb study was conducted to evaluate the safety, efficacy and pharmacokinetics of darunavir/cobicistat 800/100 mg once daily in HIV-1-infected adults with no darunavir RAMs who also received two fully active N[t]RTIs. We showed that darunavir/cobicistat 800/150 mg once daily was well tolerated, and the pharmacokinetics, virologic and immunologic responses were consistent with previously published data for darunavir/ritonavir 800/100 mg once daily.

Through Week 24, the onset of any grade 3 or 4 AEs regardless of causality was low (6% in the overall population). Five out of 313 patients (2%), all of whom were treatment-naïve, experienced any study drug-related grade 3 AE through Weeks 24 and 48. No patients experienced grade 4 drug-related AEs. The most commonly reported AEs of all grades during the study, diarrhea, nausea, upper respiratory tract infection and headache, have all been reported previously for darunavir and cobicistat [5–8, 15, 16]. Renal laboratory assessments showed small changes consistent with the inhibitory effects of cobicistat on renal tubular creatinine secretion rather than a true reduction in GFR [15, 16, 29].

Darunavir and cobicistat administered once daily with two fully active N[t]RTIs, gave a virologic response rate at Week 48 of 81% overall and 83% in treatment-naïve patients (FDA Snapshot analysis) and improved CD4^+^ cell counts over 48 weeks. The Week 48 FDA Snapshot response rate was consistent with responses using secondary analyses, including the TLOVR analysis (81% response overall and 83% in treatment-naïve patients), and with the Week 48 virologic response rate in treatment-naïve patients in ARTEMIS (84%; TLOVR, ITT analysis) [5]. As in ARTEMIS, virologic responses were similar irrespective of baseline VL.

In this study, only 15/313 patients met the criteria for resistance analysis. One of these 15 patients who was treatment experienced, developed a darunavir RAM at position I84 as a mixture with wild-type (I84I/V), which was not associated with phenotypic resistance to darunavir or other PIs. Two patients (one treatment experienced and one treatment naïve) developed the M184V RAM while receiving emtricitabine as part of their backbone N[t]RTI that was associated with phenotypic resistance to both lamivudine and emtricitabine. In the remaining 12 patients who were analyzed for resistance, no new primary RAMs were detected. The low rates of resistance development in this study confirms the high genetic barrier of darunavir whether boosted with cobicistat or ritonavir.

Steady-state darunavir pharmacokinetic parameters in the pharmacokinetic substudy were consistent with those observed in previous Phase I studies in healthy volunteers [30–32]. When data from all evaluable patients were included into a more robust dataset, the population-based darunavir pharmacokinetic parameters were consistent with previous data with darunavir/ritonavir 800/100 mg once daily in HIV-1-infected, treatment-naïve patients in ARTEMIS, and treatment-experienced patients with no darunavir RAMs in ODIN [33, 34]. In the population pharmacokinetic model, the mean darunavir C_0h_ of 2150 ng/ml was >37-fold above the protein-binding adjusted 50% effective concentration for wild-type virus (55 ng/ml) [33, 35], indicating adequate pharmacokinetic boosting of darunavir by cobicistat.

The mean steady-state cobicistat pharmacokinetic parameters (AUC_tau_ 7596 ng•h/ml and C_max_ 991 ng/ml) were consistent with levels associated with pharmacokinetic enhancement and in the range of historical data in HIV-1-infected patients (AUC_tau_ 8300 ng•h/ml and C_max_ 1100 ng/ml). Steady-state emtricitabine and tenofovir pharmacokinetic parameters were also in the range of historical data [36–38].

No clinically relevant relationship was observed between darunavir AUC_24h_ or C_0h_ and virologic response or safety at Week 24 or 48 when given with cobicistat. This is consistent with previous data with darunavir/ritonavir in ARTEMIS [33] and ODIN [34].

The fixed-dose combination of darunavir and cobicistat reduces pill burden compared with separately administered darunavir and ritonavir. Accordingly, the fixed-dose combination is expected to improve convenience and simplicity, and therefore patient adherence to medication [18–22]. Cobicistat does not induce CYP isozymes and is a more selective inhibitor of CYP3A than ritonavir [13, 14], so there is less potential for drug–drug interactions to occur than with ritonavir.

The study was limited in that it was an open-label, single-arm study. As such, it did not directly compare darunavir/cobicistat against darunavir/ritonavir. Nevertheless, previous studies have shown the fixed-dose combination of darunavir/cobicistat 800/150 mg once daily has darunavir pharmacokinetic parameters comparable to those of darunavir/ritonavir 800/100 mg once daily [30–32]. These comparable pharmacokinetic parameters are expected to translate into similar efficacy, as has been shown in an indirect comparison of darunavir/cobicistat data from the current study and combined darunavir/ritonavir data from ARTEMIS (treatment-naïve patients) and ODIN (treatment-experienced patients with no darunavir RAMs) [39].

Secondly, the study included a heterogeneous group of patients with no darunavir RAMs, consistent with the current prescribing information of darunavir 800 mg once daily; however, the majority (94%) was treatment naïve. Separate reporting of the treatment-experienced cohort would not be meaningful given the small number of these patients (n =18).

In conclusion, darunavir and cobicistat was generally well tolerated, and with a safety profile that was consistent with the one of each agent separately. The combination achieved high rates of virologic suppression over 48 weeks, with only one patient developing a darunavir RAM but without phenotypic resistance to darunavir or other PIs. The steady-state pharmacokinetic parameters for darunavir, cobicistat, emtricitabine and tenofovir following administration of darunavir and cobicistat once daily and two fully active N[t]RTIs were consistent with previously published data. These data support the use of darunavir/cobicistat in combination with N[t]RTIs for future treatment of HIV-1-infected patients with no darunavir RAMs.

## Methods

### Patients

Adult HIV-1-infected, treatment-naïve or treatment-experienced (on a stable antiretroviral regimen for ≥12 weeks prior to screening) patients with no darunavir RAMs were recruited. Patients were required to have plasma VL ≥1000 HIV-1 RNA copies/ml (Amplicor HIV-1 Monitor Test, version 1.5, Roche Diagnostics, Basel, Switzerland) at screening, eGFR_CG_ ≥80 ml/min, genotypic sensitivity to the two investigator-selected N[t]RTIs (GenoSure MG™ assay, Monogram Biosciences, South San Francisco, CA, USA), and none of the following darunavir RAMs: V11I, V32I, L33F, I47V, I50V, I54M, I54L, T74P, L76V, I84V or L89V [40]. Exclusion criteria included previous or current use of darunavir, a newly diagnosed AIDS-defining condition, proven or suspected acute hepatitis or treatment for hepatitis C, and females who were pregnant or breastfeeding.

Prior to study start, the trial protocol was reviewed and approved by an independent ethics committee or an institutional review board at each study site. The trial was conducted according to the International Conference on Harmonization guideline for Good Clinical Practice and principles of Good Clinical Practice and Declaration of Helsinki. All patients provided written informed consent.

### Study design and treatment

This was an open-label, single-arm, multicenter, 48-week, Phase IIIb trial (NCT01440569) conducted at 56 sites in the USA to evaluate the safety, efficacy and pharmacokinetics of cobicistat-boosted darunavir (as single agents) plus two fully active N[t]RTIs in HIV-1-infected adults with no darunavir RAMs. The trial consisted of a ≤35-day screening period, a 48-week treatment period and a follow-up visit 30 days after last drug intake or study discontinuation, unless patients participated in an open-label rollover study and continued receiving treatment.

Patients received darunavir 800 mg (2 x 400-mg tablets) once daily plus cobicistat (150-mg tablet) once daily, both taken with food and two N[t]RTIs, administered orally. For any patients with the M184V/I RAM present at screening, emtricitabine or lamivudine could be included as a third (not fully active) N[t]RTI for the purpose of maintaining M184V/I. Prior to Week 48, changes to the study regimen were only permitted for management of suboptimal antiviral efficacy.

An optional substudy assessed darunavir, cobicistat, emtricitabine and tenofovir pharmacokinetics. The pharmacokinetic substudy included intensive pharmacokinetic sampling over 24 hours and was performed in a subset of patients (target n =48 evaluable) at selected study sites.

### Study endpoints and assessments

The primary endpoint of the study was any treatment-emergent grade 3 (severe) or grade 4 (life threatening) AEs occurring through Week 24. Secondary outcome measures included any treatment-emergent AE through Weeks 24 and 48, including those leading to discontinuation of study drug, and antiviral efficacy at Weeks 24 and 48.

Assessments for AEs (graded according to the Gilead Sciences, Inc. Grading Scale for Severity of Adverse Events and Laboratory Abnormalities) and laboratory parameters (e.g. serum chemistry, hematology, urinalysis, eGFR_CG_, plasma VL and CD4^+^ cell count) were performed at baseline and at Weeks 2, 4, 8, 12, 16, 24, 36 and 48. A 12-lead electrocardiogram was performed at screening only. Safety analyses also included data collected on or after study drug administration through 30 days after the last dose of study drugs for patients who discontinued. AEs were coded using the Medical Dictionary for Regulatory Activities (MedDRA) Version 15.1.

Treatment adherence was assessed by median pill count, which was calculated as the number of pills taken divided by the number of pills prescribed. The proportion of patients in specified adherence categories (<90% or ≥90%) was also calculated.

HIV-1 protease and reverse transcriptase genotype/phenotype testing (PhenoSense GT™ assay, Monogram Biosciences, South San Francisco, CA, USA) was performed on samples from patients with VL ≥400 HIV-1 RNA copies/ml who had a suboptimal virologic response (VL <1 log_10_ HIV-1 RNA copies/ml reduction from baseline and ≥50 HIV-1 RNA copies/ml at the Week 8 visit, confirmed at the Week 12 visit) or who had a confirmed virologic rebound (VL <50 HIV-1 RNA copies/ml followed by a confirmed VL ≥400 HIV-1 RNA copies/ml or a >1 log_10_ HIV-1 RNA copies/ml increase in VL from the nadir) or discontinued (after Week 8) while receiving study drugs. Baseline protease/reverse transcriptase phenotyping was performed retrospectively on patients with confirmed virologic failure only if they showed evidence of reduced susceptibility to darunavir or N[t]RTIs in the background regimen.

### Pharmacokinetic measurements

For the whole study population, sparse blood samples for analysis of study drug plasma concentrations were collected over 24 hours at Weeks 2, 4, 8, 12, 16, 24, 36 and 48. For the pharmacokinetic substudy, plasma samples were collected predose, 1, 2, 3, 3.5, 4, 4.5, 5, 6, 8, 10, 12 and 24 hours post-dose at a visit between Weeks 2 and 8. Darunavir [41], cobicistat [14], emtricitabine [42] and tenofovir [42] plasma concentrations were assayed using validated liquid chromatography–mass spectrometry/mass spectrometry, with a lower limit of quantification of 5 ng/ml for darunavir, cobicistat and emtricitabine and 10 ng/ml for tenofovir.

For the pharmacokinetic substudy, steady-state pharmacokinetic parameters (C_max_, C_min_ and AUC_24h_) were obtained using non-compartmental analysis (WinNonlin^®^ software version 6.2, Pharsight Corporation, Mountain View, CA, USA). The intensive pharmacokinetic substudy data was used to revise an existing 2-compartment population pharmacokinetic model with first-order absorption [43]. The population pharmacokinetic model was then used to derive individual empirical Bayes estimates of darunavir exposure (AUC_24h_ and C_0h_) at all visits using NONMEM.

Pharmacokinetic/pharmacodynamic relationships were assessed using darunavir exposure and virologic response at Week 48. The absence or presence of select AEs (rash, diarrhea, nausea or vomiting) and worst change in laboratory parameters (alkaline phosphatase, alanine transaminase, aspartate aminotransferase, amylase, lipase, glucose, total cholesterol, low-density lipoprotein-cholesterol, high-density lipoprotein-cholesterol or triglyceride) by darunavir AUC_24h_ was evaluated.

### Data analyses

Power calculations prior to study design calculated a sample size of 300 patients would provide a 95% chance of observing ≥1 grade 3 or 4 AE if the true incident rate of the AE was 1%. This sample size would also produce a two-sided 95% CI with a half-width of 4.7% based on an assumed virologic response rate for PIs (proportion of patients with VL <50 HIV-1 RNA copies/ml) of 80%.

The ITT population included all patients who were enrolled in the study and received at least one dose of darunavir and cobicistat.

Given the association between tenofovir and renal and bone events, and that the majority of the patients were receiving this therapy, a prespecified analysis of selected renal events (Fanconi syndrome, renal failure and renal tubular disorder) and bone fractures was conducted.

The primary efficacy analysis was the virologic response rate at Week 24 or 48 by the Snapshot algorithm [44]. Virologic failure was defined as VL ≥50 HIV-1 RNA copies/ml, discontinuation of study drug prior to Week 24 or 48 due to lack of efficacy, or discontinuation due to other reasons with last available VL >50 HIV-1 RNA copies/ml. Patients with no virologic data in the Week 24 or 48 window were classified as treatment failures. Secondary efficacy analyses included virologic response rates according to the TLOVR imputation algorithm and the M = F analysis.
